# Low awareness of HPV infection and willingness of HPV vaccination among Chinese male college students in the east of China

**DOI:** 10.3389/fpubh.2022.971707

**Published:** 2022-09-20

**Authors:** Hu Ran, Yue Chen, Jun Gao, Hongxiong Guo, Shanshan Peng

**Affiliations:** ^1^Jiangsu Provincial Center for Disease Control and Prevention, Nanjing, China; ^2^Nanjing Institute of Railway Technology, Nanjing, China; ^3^Department of Hepatology, The Second Hospital of Nanjing, Nanjing, China; ^4^Nanjing University of Chinese Medicine, Nanjing, China

**Keywords:** awareness, male students, papillomavirus vaccines, willingness, vaccine hesitancy

## Abstract

**Introduction:**

Human Papillomavirus (HPV) vaccine has proven to play a major role in preventing sexually transmitted diseases and related cancers among both men and women. However, the coverage of the HPV vaccination is still limited.

**Objective:**

The study aims to evaluate the awareness of HPV and HPV vaccine, as well as the willingness to take HPV vaccine, especially factors influencing vaccination willingness among male college students.

**Methods:**

We conducted a cross-sectional investigation among male college students from six colleges in Jiangsu, China. A structured questionnaire was used to investigate the subjects' basic information, behavior habits, cognition of the HPV vaccine, and intention for HPV vaccination. Multivariate analysis modes were used to analyze the influencing factors of vaccine awareness and willingness.

**Results:**

We found that among 912 participants, only 24.34% of the participants had a “good knowledge” of HPV and HPV vaccine, and 34.54% showed a “positive attitude“ toward the HPV vaccine after obtaining knowledge of HPV and its vaccine. Factors such as immune persistence, side effects, pricing strategy, and participants' self-assessment of HPV infection were the main factors influencing the HPV vaccination.

**Conclusion:**

Strengthening health education on the HPV vaccination and finding appropriate ways to solve the problem of ”vaccine hesitancy“ will be effective in improving the coverage of the HPV vaccine and preventing related diseases. In addition, the lifting of restrictions on HPV vaccination for men in China may also prove useful.

## Introduction

Human Papillomavirus (HPV), the most common sexually transmitted infection (STI) globally, is responsible for more than 70% of cervical carcinoma, 88% of anal, 15–48% of vulvar, 78% of vaginal, and 51% of penile carcinomas (1). In China, cervical cancer accounted for as many as 110,000 cases in 2020, making it the most common malignant tumor of the female reproductive system (2, 3). However, it is worth noting that HPV infection also remains common among the male population. Centers for Disease Control and Prevention of America estimates that there are about 900 new HPV-related anal cancer cases in men each year (4). An epidemiological survey on HPV infection among male outpatients in STD clinics showed that the positive rate of HPV DNA reached 13.8% (42/305) in penis swabs in east China (5).

Fortunately, HPV vaccines are now available and have proved to play a major role in preventing sexually transmitted diseases and related cancers among both men and women (3). As for heterosexual couples, infected males may put their partners at risk of HPV and related cancers. Consequently, males who get the HPV vaccine may not only protect themselves but also protect their partners from HPV (6–8). Though mainland China has not officially approved the HPV vaccine for men, studies on this easily neglected group's awareness of HPV and willingness to vaccinate seem more necessary.

Many researchers have focused on the knowledge of HPV and attitudes to vaccination among various populations (9, 10). Taking China as an example, there have been some relevant studies based on the whole population or young women in recent years. They found their target populations had insufficient HPV-associated knowledge (4, 7, 11). However, not too many studies are willing to put effort into studying HPV vaccination intentions in young men in China mainland, partly due to the national HPV policy. A study in Hong Kong reported more than 60% of the 1,004 male college students had heard of HPV and nearly a quarter would seriously consider getting vaccinated against HPV (12). Given these realities, there seems to be a need for more research into the factors that influence HPV awareness and vaccination willingness among young males.

Young males are a group that deserves special attention, considering that they are sexually active and at a relatively high risk for HPV infection (13). Thus, the objective of this study was to figure out the knowledge and attitudes about HPV-related diseases and its vaccine as well as the intentions with regard to vaccination among male college students in Jiangsu, China.

## Methods

### Design and subjects

An observational cross-sectional study was carried out. All subjects were recruited from four undergraduate and two junior colleges located in Jiangsu Province. The investigation included male students, aged 18 years or older, enrolled in a bachelor's degree in the 2021 academic year, who voluntarily agreed to participate.

### The estimation of sample size and sampling frame

Sample size *N* is estimated according to the formula *N* = *Z*^2^*[P^*^(1–*P*)]/*E*^2^, where *Z* is the statistic, generally 1.96 (when the confidence is 95%); *E* is the error value, generally 3%; and *P* is the probability value or the incidence of the expected event. According to previous studies, it is assumed that the HPV vaccination willingness of the target population (*P*) is about 30% (14–16). When *Z* = 1.96, *E* = 3%, and *P* = 0.3, the sample size was calculated and then determined to be 896 at least. Considering that there may be invalid questionnaires, the sample size was increased by 10% correspondingly. Finally, a total of 985 electronic questionnaires were distributed to the target population. We numbered all the male students in these six colleges and arranged them in order. Sampling was conducted using the random number table method.

### The design of the questionnaire

The questionnaire consists of 26 items structured into three sections (Supplement 1). The first section consists of five items, including sociodemographic data and personal information. The second section consists of 15 dichotomous items, including knowledge of HPV infection and its vaccination. The awareness rate is defined as the number of respondents who have a good knowledge of HPV and HPV vaccine (answered 9 or more of the 15 questions correctly) divided by the number of respondents. After this section, students will be given publicity material such as a promotional video about HPV and its vaccine before they can proceed to the third section, which assesses their attitudes toward HPV vaccination. The questionnaire partly refers to Mohammed Dany's and Sara Villanueva's research (10, 17).

An invalid questionnaire was defined with any one situation among the following: (1) more than two-thirds of the questions are not answered; (2) all options selected in the questionnaire are the same, such as ”A“; (3) the options in the whole questionnaire are regularly answered in recurring patterns. All investigations were submitted online after informed consent was obtained.

To better test the reliability and feasibility of the questionnaire, we conducted a pre-test on the questionnaire. Eighty students were randomly selected, and investigated according to the formal survey. We completed the reliability and validity analysis by SPSS 22.0. Cronbach's alpha coefficients are ≥0.6, which showed the reliability of the questionnaire is qualified.

The questionnaires were anonymous and self-completed between February and May 2021. Students were free to omit any questions that they did not want to answer. To get high participation levels, we trained the related school administrators and provided appropriate funds for the project. These school administrators uniformly distributed electronic questionnaires to the selected subjects and stressed the importance of the project.

### Statistical analysis

According to data type and distribution, percentage and percentage of (x ± s) or median (quad interval) were used for statistical description. Logistic univariate regression was used to compare the differences in HPV cognition and vaccination willingness of the subjects with different socio-demographic characteristics. The variables *P* < 0.1 in the univariate test were included in the logistic multivariate regression model. *P* < 0.05 was considered statistically significant.

## Results

### Demographic characteristics

A total of 912 male college students submitted their valid questionnaires online (response rate of 92.6%). The remaining students did not answer the questionnaire online or their questionnaires were invalid. All the students who submitted the valid questionnaires answered all the questions. Table 1 shows the demographic characteristics of the subjects. The average age of the subjects was 20.42 ± 0.73 years. Of them, 61.84% (564/912) are registered in rural regions, 43.09% (393/912) are studying a medical major at a medical university, and 43.75% (399/912) are studying as junior college students (shown in Table 1).

**Table 1:** Demographic characteristics of all subjects.

**Characteristics**		***N* (%)**
Registered residence	Rural	564 (61.84)
	Urban	348 (38.16)
Major	Non-medicine	519 (56.91)
	Medicine	393 (43.09)
School category	Junior college	513 (56.25)
	Undergraduate college	399 (43.75)
Sexual orientation	Heterosexuality	844 (92.54)
	Homosexuality/bisexuality	68 (7.46)
Number of sexual partners in the past 24 months	0–1	804 (88.16)
	2 or more	108 (11.84)
Based on my lifestyle, I believe that I am susceptible to the HPV infection and must get the vaccine	No	690 (75.66)
	Yes	222 (24.34)
I have faith in vaccine immune persistence	No	795 (87.17)
	Yes	117 (12.83)
I believe that the current HPV vaccine is capable of preventing the occurrence of cervical cancer	No	739 (81.03)
	Yes	173 (18.97)
I believe that the current HPV vaccine is safe enough	No	489 (53.62)
	Yes	423 (46.38)
I believe that the price of the vaccine is affordable, given the benefits it offers	No	567 (62.17)
	Yes	345 (37.83)

### Awareness of HPV infection and HPV vaccination

Of the 912 students, 222 answered 9 or more of the 15 questions correctly, making the awareness rate of HPV reach 24.34%. Quite a number of male students knew about the main routes of infection, even the association of HPV with cervical cancer and genital warts; however, they failed to realize the HPV vaccine is actually available abroad for men and protects against some sexually transmitted diseases (as in Table 2).

**Table 2:** Knowledge of HPV infection and vaccination.

**Item (Correct answer)**	**Correct *N* (%)**	**Incorrect *N* (%)**	**DK/NO *N* (%)**
1. The type of cancer highly associated with HPV infection is uterine cervical cancer (true)	733 (80.37)	107 (11.73)	72 (7.9)
2. Human papillomavirus can cause herpes (false)	98 (10.75)	443 (48.57)	371 (40.68)
3. Human papillomavirus can lead to genital warts (growths on the skin of the genitals) (true)	521 (57.13)	9 (0.99)	382 (41.88)
4. HPV can be transmitted through vaginal, anal, and oral sex as well as genital to genital contact (true)	809 (88.71)	89 (9.76)	14 (1.53)
5. In most cases, HPV-infected women do not show symptoms (true)	595 (65.24)	91 (9.98)	226 (24.78)
6. All HPV infections are caused by the same type of virus (false)	411 (45.06)	209 (22.92)	292 (32.02)
7. HPV-positive pregnant women can pass the virus to their babies (false)	90 (9.87)	705 (77.3)	117 (12.83)
8. Only females can be infected with HPV and show symptoms (false)	334 (36.62)	543 (59.54)	35 (3.84)
9. HPV can be transmitted from a carrier to his/her partner only if the carrier shows symptoms (false)	234 (25.66)	471 (51.64)	207 (22.7)
10. There is no current cure or therapy for HPV infection (true)	276 (30.26)	398 (43.64)	238 (26.1)
11. HPV vaccines have the same effect whether the female takes it before or after being infected with HPV (false)	645 (70.72)	91 (9.98)	176 (19.3)
12. HPV vaccine is best taken before starting to have sexual activities (true)	398 (43.64)	22 (2.41)	492 (53.95)
13. HPV vaccine can only be taken after the age of 16 years (false)	465 (50.99)	427 (46.82)	20 (2.19)
14. The HPV vaccine is banned for men in China (false)	887 (97.26)	12 (1.31)	13 (1.43)
15. The HPV vaccine has no effect for men (false)	278 (30.48)	561 (61.51)	73 (8.01)

To know the association of demographic characteristics with the awareness of HPV and HPV vaccination, “good knowledge” was taken as the dependent variable to conduct logistic univariate analysis. Variables that were statistically significant in univariate analysis or found to be significant in the published literature were included in the multivariate analysis (as shown in Table 3). Awareness of HPV in students majoring in medicine or students with homosexual or bisexual sexual orientation was higher than in their counterparts (*P* < 0.001).

**Table 3:** Logistic multivariate analysis of HPV vaccine awareness rate among male college students in Nanjing, China.

**Characteristics**	**Distribution**	**Number**	**People who have good knowledge of HPV & HPV vaccine *N* (%)**	***p*-value**	**OR (95% CI)**
Birth place	Rural	564	131 (23.23)		1
	Urban	348	91 (26.15)	0.053	1.453 (0.996–2.212)
Major	Non-medicine	519	92 (17.73)		1
	Medicine	393	130 (33.08)	<0.001	2.242 (1.608–3.126)
School category	Junior college	513	112 (21.83)		1
	Undergraduate college	399	110 (27.57)	0.668	0.923 (0.639–1.332)
Sexual orientation	Heterosexuality	844	183 (21.68)		1
	Homosexuality/bisexuality	68	39 (57.35)	<0.001	3.655 (2.09–6.392)

### Willingness of HPV vaccination

To know the association of demographic characteristics with HPV infection and the willingness of HPV vaccination, the variable “positive attitude” was taken as the dependent variable to conduct a logistic univariate analysis. Variables that were statistically significant in univariate analysis or found to be significant in the published literature were included in the multivariate analysis (as shown in Table 4).

**Table 4:** Logistic multivariate analysis of HPV vacci nation willingness among male college students in Nanjing, China.

**Characteristics**	**Distribution**	**Number**	**People who are willing to be vaccinated with HPV vaccine *N* (%)**	***p*-value**	**OR (95% CI)**
Birth place	Rural	564	124 (21.99)		1
	Urban	348	191 (54.89)	<0.001	6.082 (3.782–9.780)
Major	Non-medicine	519	138 (26.59)		1
	Medicine	393	177 (45.04)	0.543	1.152 (0.731–1.817)
School category	Junior college	513	91 (17.74)		1
	Undergraduate college	399	224 (56.14)	<0.001	3.719 (2.359–5.861)
Sexual orientation	Heterosexuality	844	250 (29.62)		1
	Homosexuality /bisexuality	68	65 (95.59)	<0.001	84.381 (21.365–333.264)
Number of sexual partners in the past 24 months	0–1	804	259 (32.21)		1
	2 or more	108	56 (51.85)	0.198	1.59 (0.785–3.22)
Based on my lifestyle, I believe that I am susceptible to the HPV infection and must get the vaccine	No	690	167 (54.20)		1
	Yes	222	148 (66.67)	<0.001	5.77 (3.278–10.157)
I have faith in vaccine immune persistence	No	795	220 (27.67)		1
	Yes	117	95 (81.20)	0.001	3.104 (1.545–6.238)
I believe that the current HPV vaccine is capable of preventing the occurrence of cervical cancer	No	739	195 (26.39)		1
	Yes	173	120 (69.36)	0.142	1.519 (0.87–2.652)
I believe that the current HPV vaccine is safe enough	No	489	146 (29.86)		1
	Yes	423	169 (39.95)	0.022	1.73 (1.084–2.762)
I believe that the price of the vaccine is affordable, given the benefits it offers	No	567	104 (18.34)		1
	Yes	345	211 (61.16)	<0.001	8.264 (5.335–12.801)

Before popularizing the knowledge of HPV vaccine preventing diseases, only 87 students, accounting for 9.54% of the total number of respondents, had the intention of HPV vaccination. After popularizing the knowledge related to HPV and its vaccine, 315 respondents had the intention of HPV vaccination, accounting for 34.54% of the total number of respondents. In addition, statistical analysis showed that the main reasons for male college students' reluctance to receive the HPV vaccine were their doubts about the safety and immune persistence of the vaccine, their assessment of their own susceptibility to HPV infection, as well as their perception of the high price of the vaccine.

## Discussion

In this study, we explored the level of knowledge about HPV infection and attitude toward the HPV vaccine among male college students in Jiangsu, China. Only 24.34% showed good knowledge of HPV and its vaccine. After learning about relevant knowledge of HPV and the HPV vaccine, not many respondents (34.54%) expressed a high interest in getting vaccinated against HPV. HPV awareness rate and vaccination willingness rate are close to similar studies at home and abroad (4, 7, 11, 14, 15). We believe both rates among the male general population may be lower than the target population of this study. After all, university-age males are more receptive.

A survey implemented by Tsinghua University showed that male college students in China tend to have insufficient knowledge about HPV and the HPV vaccine compared with their counterparts in some developed countries (15). Our study confirms their findings. This may be partly due to the lack of HPV-related health education or sex education in China (18), or the fact that Chinese college students are shy about discussing sex (19).

Similar to some other studies, the majority correctly recognized that HPV is the cause of uterine cervical cancer. Most students knew the main transmission routes of HPV, but quite a few mistakenly believed that an HPV-positive pregnant woman can pass the virus to their babies (10, 20). This shows that some of the students may not have a systematic understanding of HPV and related diseases, but only get some knowledge from fragmentary information.

An answer that also caused us great concern was the high percentage of college male students who failed to recognize that HPV infection could be contracted by men just as easily as by women. Moreover, many male college students are skeptical about HPV vaccination against men, believing that this vaccine may be of no value to men. A study in Spain suggested it may be related to the national HPV policy (10). For example, Italy's free HPV vaccine is available to boys and girls, while in Spain it is only free for girls. Parents in Spain will have to buy the HPV vaccine if they want their sons to have it. Therefore, Spanish researchers believed that this is the reason for the cognitive difference between the target populations of the two countries (10). We basically agree with this view. The national HPV policy in mainland China claims expressly that the HPV vaccine can only be given to women. In addition, approval for males still seems light years away. This difference in policy may lead to a lack of awareness of HPV infection and HPV vaccination for men (21, 22).

It should be noted that the medical students' awareness of HPV and its vaccine is significantly higher than in other majors. However, after providing all the participants with information about the virus and the vaccine, 45.04% of the medical students were willing to receive HPV vaccination, no difference from other majors. The finding suggested that vaccination intention might be also influenced by economic, policy, health consciousness, and other factors as well as the level of medical knowledge. This gives us a direction for the effectiveness of health education measures, not only to improve students' knowledge level but also to change their attitudes (15).

Despite being recognized as the only vaccine against cancer, HPV vaccination is perceived as unsafe and unnecessary by some students. One of the main reasons why vaccine coverage is limited is attributable to vaccine hesitancy. The determinants of individual decision-making about vaccination included but are not limited to vaccine safety, vaccine effectiveness and vaccine immune persistence, and price of the vaccine. Many experts had proposed ways to deal with the problems of vaccine hesitancy, including providing information about the susceptibility and severity of the disease and the effectiveness and safety of the vaccine (23, 24). Larson was inclined to stress that the authorities should place additional emphasis on listening to the concerns and understanding the perceptions of the public, and publishing the vaccine marketing authorization process and post-marketing surveillance data, not just considering transparency in policy-making decisions on vaccination programs (25, 26).

There are some limitations to this study. First, the target population of the study is college students, who have an insufficient economic source. There is no doubt that the price of vaccines may influence their willingness of HPV vaccination. Second, the schools selected by the institute should have been randomly selected from all schools in East China. However, only six schools were selected, which may lead to selection bias.

In light of the present findings, it is suggested that strengthening health understanding about HPV and its vaccine is most needed among male college students. However, increased awareness only partially increases the willingness to vaccinate. To significantly increase vaccine coverage, the long-standing problem of vaccine hesitancy must be investigated from a different perspective. In addition, HPV has already been shown to infect both men and women, causing sexually transmitted diseases and cancers; primary prevention such as HPV vaccination should be done for both genders. Given China's huge population, free HPV vaccination may not be a realistic policy, but lifting restrictions on men would also work well.

## Conclusion

The awareness of HPV infection and willingness of HPV vaccination are low among Chinese male college students. This can be increased by popularizing the knowledge of the HPV vaccine to prevent the disease. The hesitation on vaccine's safety, higher price, and current HPV vaccine policy also decreased the willingness of HPV vaccination in the male population. To reduce public concern caused by HPV-related disease, it is necessary to publicize more widely the corresponding knowledge linked to HPV infection and vaccine in the male population and adjust vaccination policy.

## Data availability statement

The original contributions presented in the study are included in the article/Supplementary material, further inquiries can be directed to the corresponding authors.

## Ethics statement

The studies involving human participants were reviewed and approved by the Ethics Committee of Jiangsu Provincial Center for Disease Control and Prevention. The patients/participants provided their written informed consent to participate in this study.

## Author contributions

HR and HG designed the questionnaire, analyzed data, and drafted the manuscript. JG, SP, and YC conducted the investigation. All authors contributed to the article and approved the submitted version.

## Funding

This project was supported partly by the project funded by the Scientific Research Project of the Jiangsu Provincial Health Commission (M2021030).

## Conflict of interest

The authors declare that the research was conducted in the absence of any commercial or financial relationships that could be construed as a potential conflict of interest.

## Publisher's note

All claims expressed in this article are solely those of the authors and do not necessarily represent those of their affiliated organizations, or those of the publisher, the editors and the reviewers. Any product that may be evaluated in this article, or claim that may be made by its manufacturer, is not guaranteed or endorsed by the publisher.
