# Supplementary material for: Low awareness of HPV infection and willingness of HPV vaccination among Chinese male college students in the east of China
Source: Front Public Health. 2022 Sep 20;10:971707. doi: 10.3389/fpubh.2022.971707 (PMC9531242; doi:10.3389/fpubh.2022.971707)
Supplement: Supplementary file 1 [file Data_Sheet_1.doc]

**Human papillomavirus (HPV) survey**

Dear students, thank you for taking time out of your busy schedule to participate in this survey. This survey is aimed at your knowledge of HPV and vaccine as well as your willingness to be vaccinated. Thank you again for taking up your valuable time. Please choose the answer you think appropriate to the following questions.

1. Personal Characteristics

| 1. 1 Birth place A.(Rural);B(Urban) |
| --- |
| 1.2 Major A.(Non-Medicine); B.(Medicine) |
| 1.3 School category A.(Junior college); B(Undergraduate College) |
| 1.4 Sexual orientation A.(Heterosexuality); B(Homosexuality/bisexuality) |
| 1.5 Number of sexual partners in the past 24 months A.(0-1); B.(2 or more) |

1. Awareness and Knowledge of HPV Infection

| 2.1 The type of cancer highly associated with HPV infection is uterine cervical cancer (true or false) |
| --- |
| 2.2 Human papillomavirus can cause herpes (true or false) |
| 2.3 Human papillomavirus can lead to genital warts (growths on the skin of the genitals) (true or false) |
| 2.4 HPV can be transmitted through vaginal, anal, and oral sex as well as genital to genital contact (true or false) |
| 2.5 In most cases, HPV-infected women do not show symptoms (true or false) |
| 2.6 All HPV infections are caused by the same type of virus (true or false) |
| 2.7 HPV-positive pregnant women can pass the virus to their babies (true or false) |
| 2.8 Only females can be infected with HPV and show symptoms (true or false) |
| 2.9 HPV can be transmitted from a carrier to his/her partner only if the carrier shows symptoms (true or false) |
| 2.10 There is no current cure or therapy for HPV infection (true or false) |
| 2.11 HPV vaccines have the same effect whether the female takes it before or after being infected with HPV (true or false) |
| 2.12 HPV vaccine is best taken before starting to have sexual activities (true or false) |
| 2.13 HPV vaccine can only be taken after the age of 16 years (true or false) |
| 2.14 The HPV vaccine is banned for men in China (true or false) |
| 2.15 The HPV vaccine has no effect for men (true or false) |

1. Attitude towards HPV vaccination

| 3.1 Are you willing to receive the HPV vaccine if it is licensed for males in mainland China?  (yes or no)  If you checked “No”, please answer the following questions |
| --- |
| 3.2 Based on my lifestyle, I believe that I am susceptible to the HPV infection and must get the vaccine.(yes or no) |
| 3.3 I have faith in vaccine immune persistence.(yes or no) |
| 3.4 I believe that the current HPV vaccine is capable of preventing the occurrence of cervical cancer.(yes or no) |
| 3.5 I believe that the current HPV vaccine is safe enough.(yes or no) |
| 3.6 I believe that the price of the vaccine is affordable, given the benefits it offers.(yes or no) |
